# Low rate of positive margins and re-excision after partial mastectomy in highly selected breast cancer patients: A Chinese single-institution experience

**DOI:** 10.18632/oncotarget.14686

**Published:** 2017-01-17

**Authors:** Siyu Wu, Yanyan Zhu, Zhaozhi Yang, Miao Mo, Hongbo Gao, Wentao Yang, Guangyu Liu

**Affiliations:** ^1^ Department of Breast Surgery, Fudan University Shanghai Cancer Center, Department of Oncology, Shanghai Medical College, Fudan University, Shanghai, P.R. China; ^2^ Department of General Surgery, Jinshan Hospital, Fudan University, Shanghai, P.R. China; ^3^ Department of Radiation Oncology, Fudan University Shanghai Cancer Center, Department of Oncology, Shanghai Medical College, Fudan University, Shanghai, P.R. China; ^4^ Clinical Statistics Center, Fudan University Shanghai Cancer Center, Department of Oncology, Shanghai Medical College, Fudan University, Shanghai, P.R. China; ^5^ Department of Radiology, Fudan University Shanghai Cancer Center, Department of Oncology, Shanghai Medical College, Fudan University, Shanghai, P.R. China; ^6^ Department of Pathology, Fudan University Shanghai Cancer Center, Department of Oncology, Shanghai Medical College, Fudan University, Shanghai, P.R. China

**Keywords:** low rate, positive margins, highly selected, partial mastectomy, Chinese

## Abstract

A recent randomized controlled trial firstly demonstrated that cavity shaving significantly decreased the rate of positive margins and re-excision among partial mastectomy (PM) patients. However, it remains unknown whether cavity shaving should be routinely applied to Chinese breast cancer patients undergoing PM. A total of 408 PM patients undergoing 410 PMs among 1796 surgically treated breast cancer patients at Fudan University Shanghai Cancer Centre from January 2015 to June 2015 were included in our study. Data were analysed with univariate or multivariate analysis. Overall, 11 of 410 cases (2.7%) had positive margins postoperatively. Moreover, only 24.6% of the cases (*P*<0.05) presented with ductal carcinoma *in situ* (DCIS), among whom 10.0% obtained positive margins. In multivariate logistic regression analysis, presence of mammographic calcifications was significantly associated with margin positivity (P<0.05, OR=6.06, 95% CI: 1.53-23.91). In conclusion, cavity shaving during PM should not be routinely performed in Chinese breast cancer patients, particularly in highly selected cases with a low prevalence of DCIS. PM patients with preoperative mammographic calcifications were more likely to have positive margins and might benefit more from cavity shaving.

## INTRODUCTION

A series of important randomized controlled trials (RCTs) consistently demonstrated that partial mastectomy (PM) plus radiation therapy could achieve overall survival rates similar to mastectomy while improving patients’ aesthetic and psychological outcomes established standard status of PM for early breast cancer [1, 2].

In contrast to mastectomy patients, PM patients are more likely to develop local recurrence. The latest meta-analysis, EBCTCG in 2011, revealed that for PM patients, one cancer-related death could be avoided over 15 years for every 4 patients with breast cancer recurrence over 10 years [3, 4]. Additionally, further re-excision was always required subsequently, which would undoubtedly increase the patient burden regarding economic considerations and cosmetic outcomes. Obtaining a negative surgical margin, one of the strongest predictors of lack of tumour recurrence, is of great clinical benefit and should be achieved to ensure an expectably low rate of local recurrence after PM.

Recently, Chagpar et al. established that PM patients with cavity shaving have a remarkably decreased rate of positive margins and re-excision in a RCT [5], thus bringing an end to years of retrospective analyses regarding cavity shaving. However, whether cavity shaving should be routinely conducted in the context of a Chinese population remains an unaddressed issue. We sought to address the feasibility of margin shaving in Chinese breast cancer patients in this study describing a Chinese single-institution experience.

## RESULTS

A total of 408 patients undergoing 410 PMs from January 2015 to June 2015 were enrolled in this retrospective single-institutional study.

### Clinicopathological characteristics

In our cohort, the median age was 46 years (range 19-82 years), and the median tumour size was 1.5 cm. 350 of 410 patients (85.4%) had palpable tumors preoperatively and DCIS components were present in 101 patients postoperatively, accounting for 24.6% of all PM patients (Table 1).

**Table 1:** Clinicopathological characteristics of patients who underwent initial breast-conserving surgery categorised by margin status

Variable	N (Percent)	Margin status		*P* value
		Positive	Negative	
**Age (years)**				>0.05
** Median**	46	42	46	
** Range**	19-82	38-77	19-82	
** ≤40**	105 (25.6%)	3 (2.9%)	102 (97.1%)	
** >40**	305 (74.4%)	8 (2.6%)	297 (97.4%)	
**Palpable tumor**				>0.05
** Yes**	60 (14.6%)	3 (5%)	57 (95%)	
** No**	350 (85.4%)	8 (2.3%)	342 (97.7%)	
**Mammographic calcifications**				<0.05
** Yes**	110 (26.8%)	7 (6.4%)	103 (93.6%)	
** No**	280 (68.3%)	4 (1.4%)	276 (98.6%)	
** NA**	20 (4.9%)	0 (0%)	20 (100%)	
**Neo-adjuvant therapy**				>0.05
** Yes**	41 (10%)	0 (0%)	41 (100%)	
** No**	369 (90%)	11 (3.0%)	358 (97.0%)	
**Histologic type**				
** Ductal**	373 (91.0%)	9 (2.4%)	364 (97.6%)	
** Lobular**	9 (2.2%)	0 (0%)	9 (100%)	
** Other**	21 (5.1%)	2 (9.5%)	19 (90.5%)	
** NA**	7 (1.7%)	0 (0%)	7 (100%)	
**Presence of DCIS**				<0.05
** Yes**	101 (24.6%)	10 (10.0%)	91 (90.0%)	
** No**	309 (75.4%)	1 (0.3%)	308 (99.7%)	
**Tumour size (cm)**				>0.05
** Median**	1.5	1.2	1.5	
** Range**	0-4	1-3.5	0-4	
** ≤2**	331 (80.7%)	8 (2.4%)	323 (97.6%)	
** >2**	76 (18.5%)	2 (2.6%)	74 (97.4%)	
** NA**	3 (0.8%)	1 (33.3%)	2 (66.7%)	
**Lymph node status**				>0.05
** Positive**	93 (22.7%)	1 (1.1%)	92 (98.9%)	
** Negative**	313 (76.3%)	10 (3.2%)	303 (96.8%)	
** NA**	4 (1.0%)	0 (0%)	4 (100%)	
**Lymphovascular invasion**				>0.05
** Positive**	78 (19.0%)	1 (1.3%)	77 (98.7%)	
** Negative**	297 (72.4%)	10 (3.4%)	287 (96.6%)	
** NA**	35 (8.5%)	0 (0%)	35 (100%)	
**Histological grade**				>0.05
** 1**	12 (2.9%)	0 (0%)	12 (100%)	
** 2**	148 (36.1%)	2 (1.4%)	146 (98.6%)	
** 3**	140 (34.1%)	4 (2.9%)	136 (97.1%)	
** High (pure DCIS)**	13 (3.2%)	1 (7.7%)	12 (92.3%)	
** Median (pure DCIS)**	18 (4.4%)	1 (5.6%)	17 (94.4%)	
** Low (pure DCIS)**	12 (2.9%)	0 (0%)	12 (100%)	
** Other**	67 (16.3%)	3 (4.5%)	64 (95.5%)	
** NA**	11 (2.7%)	0 (0%)	11 (100%)	
**ER status**				>0.05
** Negative**	111 (27.1%)	4 (3.6%)	105 (96.4%)	
** Positive**	297 (72.4%)	7 (2.4%)	290 (97.6%)	
** NA**	2 (0.5%)	0 (0%)	2 (100%)	
**PR status**				>0.05
** Negative**	133 (32.4%)	7 (5.3%)	126 (94.7%)	
** Positive**	275 (67.1%)	4 (1.5%)	271 (98.5%)	
** NA**	2 (0.5%)	0 (0%)	2 (100%)	
**HER-2 status**				>0.05
** Negative**	330 (80.5%)	9 (2.7%)	321 (97.3%)	
** Positive**	76 (18.5%)	2 (2.6%)	74 (97.4%)	
** NA**	4 (1.0%)	0 (0%)	4 (100%)	
**Ki-67**				>0.05
** Low (≤20%)**	204 (49.8%)	6 (2.9%)	198 (97.1%)	
** High (>20%)**	201 (49.0%)	5 (2.4%)	196 (97.6%)	
** NA**	5 (1.2%)	0 (0%)	5 (100%)	

NA: not accessed; DCIS: ductal carcinoma *in situ*; ER: oestrogen receptor; PR: progesterone receptor; HER-2: human epidermal growth factor receptor-2.

A total of 11 patients had positive margins in the final pathological findings (Table 1); of these patients, 6 underwent re-excision, including 4 who were converted to mastectomy and 2 with positive margins who required further resection. The remaining patients did not undergo further surgery. A significant difference was observed between patients with positive and negative margins in terms of the presence of mammographic calcifications (yes or no) as well as DCIS components.

### Univariate and multivariate analysis for positive margins

Univariate analyses revealed that the presence of mammographic calcifications as well as presence of DCIS. A series of important randomized controlled trials (RCTs) were risk factors significantly associated with positive margins in the first cohort (Table 1, 2).

**Table 2:** Univariate and multivariate analysis for the factors of positive margins

Factors	Univariate		Multivariate	
	OR(95%CI)	*P* values	OR (95%CI)	*P* values
**Age(years)**				
** ≤40**	1.00		1.00	
** >40**	0.92 (0.24-3.50)	0.90	0.67 (0.16-2.78)	0.59
**Tumour size(cm)**				
** ≤2**	1.00		1.00	
** >2**	0.99 (0.21-4.76)	0.99	0.81 (0.16-4.23)	0.80
**Mammographic calcifications**				
** No**	1.00		1.00	
** Yes**	3.97 (1.14-13.80)	0.03	6.06 (1.53-23.91)	0.01
**Lymphovascular invasion**				
** No**	1.00		1.00	
** Yes**	0.42 (0.05-3.39)	0.42	0.47 (0.05-4.52)	0.51
**Lymph node status**				
** Negative**	1.00		1.00	
** Positive**	0.33 (0.04-2.61)	0.29	0.32 (0.04-2.59)	0.29
**ER status**				
** Negative**	1.00		1.00	
** Positive**	0.64 (0.18-2.22)	0.48	0.483 (0.13-1.81)	0.28
**HER-2 status**				
** Negative**	1.00		1.00	
** Positive**	0.95 (0.20-4.49)	0.95	0.755 (0.15-3.86)	0.74
**Ki-67**				
** ≤20%**	1.00		1.00	
** >20%**	0.68(0.19-2.44)	0.55	0.874 (0.19-4.10)	0.86

### Multivariate analysis for positive margins

Notably, for 110 patients presenting with and 280 patients without mammographic calcifications, the percentages of patients who presented with DCIS components were 37.3% and 19.3%, respectively (*P*<0.05) (Table 1). Considering that it was widely accepted that such a significant correlation between DCIS and mammographic calcifications as present in our study as well as previous reports, and diagnosis of DCIS was mostly obtained postoperatively, instead of DCIS, presence of preoperative mammographic calcifications was incorporated into multivariate analysis. As was shown in Table 2, multivariate analysis showed that the presence of mammographic calcifications was significantly associated with positive margins after PM (*P*=0.01, OR=6.06, 95%CI: 1.53-23.91).

In addition, Table 3 showed the correlations between the features of mammographic calcifications and margin status in 96 patients whose information was available. However, no significant differences were observed between the calcification features including type, morphology, distribution, range and margin status. It was noted that only 4.9% of these patients had calcifications in the range of >30 mm.

**Table 3:** Correlations between calcification features and margin status

		N=96	Margin status		*P* value
			Positive	Negative	
**Calcification type**	Calcifications only	38 (39.6%)	3 (7.9%)	35 (92.1%)	>0.05
	Calcifications with mass	33 (34.4%)	3 (9.1%)	30 (90.9%)	
	Calcifications with asymmetric compactness	10 (10.4%)	1 (10.0%)	9 (90.0%)	
	Calcifications with architectural distortion	15 (15.6%)	0 (0%)	15 (100%)	
**Morphology**	Fine branching	94 (97.9%)	7 (7.4%)	87 (92.6%)	>0.05
	Pleomorphic	2 (2.1%)	0 (0%)	2 (100%)	
**Distribution**	Clustered	50 (52.1%)	6 (12.0%)	44 (88.0%)	>0.05
	Regional	4 (4.2%)	0 (0%)	4 (100%)	
	Linear	9 (9.4%)	1 (11.1%)	8 (88.9%)	
	Segmental	33 (34.4%)	0 (0%)	33 (100%)	
**Range**	≤20 mm	69 (71.9%)	6 (8.7%)	63 (91.3%)	>0.05
	>20 mm	13 (13.5%)	1 (7.7%)	12 (92.3%)	
	NA	14 (14.6%)	0 (0%)	14 (100%)	

### Change in the rate of PM in the past five years

From January 2010 to June 2015, the PM rate rose steadily at our centre, increasing from 18.1% to 22.7%, which is the highest PM rate at our centre during the past 5 years (Supplementary Table 1).

## DISCUSSION

A recent RCT concluded that a significant reduction in the rate of positive margins and re-excision was demonstrated by PM patients with cavity shaving, bringing level I evidence to cavity shaving after years of persistent debate and changing the surgical management of PM treatment [6–8]. However, our study demonstrated that cavity shaving should not be routinely conducted in highly selected Chinese breast cancer patients due to the low rate of positive margins after an initial PM.

The highlights of our study include that it is the first study to investigate the feasibility of applying cavity shaving in Chinese breast cancer patients, and it further explored the associations among positive margins and highly selected PM patients in a Chinese population compared to foreign countries. Collectively, our results showed that cavity shaving should not be performed in highly selected Chinese breast cancer patients who undergo a PM.

First, it was noted that PM accounted for only 22.7% of the surgical procedures for primary breast cancer, in contrast to 60-70% in the US [9]. This finding is also consistent with our previous data obtained during the past 5 years and, more importantly, with countrywide data reporting a PM rate of 11.9% [10]. In addition to the shortage of radiation equipment noted in Fan L's review [11] and the relatively small breast volume of Chinese women [12, 13], this large difference may be explained by the strict PM criteria commonly adopted at our centre and in China. We diligently eliminated patients with diffuse or extensive mammographic calcifications preoperatively, which might have partly contributed to the high selectivity for patients receiving a PM in our centre, thus the low rate of positive margins and re-excision. However, in the NCCN guideline, this is not an absolute PM contraindication in foreign countries where a second or even a third re-excision is allowed [14].

Chagpar at el. found that the presence and size of DCIS were both significantly associated with margin positivity [5]. Accordingly, the percentage of patients with DCIS components in the current study was only 24.6%, which was significantly lower than the value of 72.3% in Chagpar's study [5], firmly illustrating that the high selectivity of PM patients employed in our study produced a low rate of positive margins.

As a result, in contrast to the 2.7% margin positivity following the first attempted PM in our study, a significantly higher rate of positive margins even after performing cavity shaving (19%) was reported in the trial [5]. Similarly, the percentage of patients with positive margins following initial PM also ranged from 20% to 40% in most previous analogous studies [15]. More importantly, an observational study based on a large population of 2206 patients in the US reported a 12.1% positive margin (0.0-0.9 mm) rate along with a rate of 22.9% for re-excision of the initial PM [14].

Of note, the existing phenomenon might also be largely attributed to the substantial differences in conditions and culture between China and other countries such as insurance, traditions, value concepts and other factors. Re-excision signifies re-hospitalization and undoubtedly an increased expense for patients and constitutes a psychological burden for surgeons [14]. Consequently, unlike our hospital, most hospitals in China choose intraoperative frozen section evaluation, as reported in the study by Chen K [16]. Therefore, a more conservative standard for PM was justifiably adopted at our centre because intraoperative pathological evaluation was too time-consuming to meet the demands of the increasing numbers of breast cancer patients treated at our centre every year from 1833 cases in 2010 to 3678 cases in 2014.

Given that it has been well accepted that most DCIS patients present with calcifications on preoperative mammography [17], we made several interesting observations. In our study, patients with calcifications were indeed more likely to present with DCIS. Correspondingly, a higher proportion of PM patients with palpable tumours than in Chagpar's study also indirectly indicated that fewer patients might have presented with preoperative mammographic calcifications in our study cohort. Most importantly, we also found that the presence of mammographic calcifications (yes or no) was significantly associated with positive margins (OR=6.1, P<0.05). In our experience, surgeons have used caution in choosing patients with calcifications for PM, mostly choosing PM for calcifications ≤20 mm and particularly excluding those patients whose calcifications were >30 mm. This observation was also corroborated by the study data in that the patients with calcifications >20 mm and >30 mm accounted for only 15.9% and 4.9%, respectively, of the total study population.

It was noteworthy that on the basis of such a low rate of positive margins after PM, some previous work regarding PM conducted in our centre revealed, to some extent, that an acceptable local control as well as overall survival could be obtained, despite of different patient population [18, 19].

Finally, we sought to analyse the associations between the features of the calcifications and margin status. No significant correlations were found between these factors, which might be attributable to the limited number of patients with positive margins. Therefore, this topic will be a possible focal point in the next investigative stage.

Therefore, taken together, we have indicated that cavity shaving should not be routinely performed in highly selected Chinese breast cancer patients with a low rate of DCIS who receive a PM. Moreover, PM patients with mammographic calcifications might tend to be present with DCIS, thus constituting a specific target population with a higher risk of positive margins. Intuitively but hypothetically, this specific group of PM patients might have undergone cavity shaving among the population of PM patients with higher margin positivity who were not highly selected preoperatively, such as those in Chagpar's and other foreign studies. However, this assumption should be further investigated in RCTs due to the lack of a direct comparison.

In retrospect, the limited number of patients with positive margins and missing data also lowered the power of the study conclusions. Moreover, the retrospective nature, which could result in various biases, was also an inherent disadvantage.

In conclusion, cavity shaving during PM should not be conducted in Chinese breast cancer patients with a low prevalence of DCIS. Patients with preoperative mammographic calcifications were more likely to have positive margins and, hypothetically, might have indications for cavity shaving during PM in foreign countries, which deserves further investigation in RCTs.

## MATERIALS AND METHODS

### Patients

The inclusion criteria for this retrospective study encompassed the patients undergoing PM at Fudan University Shanghai Cancer Centre (FUSCC). The eligibility for PM was assessed by the surgeons following strict adherence to the relevant guidelines, that is, T1-T2 stage with an expected good cosmetic outcome after PM or meeting such criteria after neo-adjuvant therapy. The absolute exclusion criteria encompassed patients with contraindications for radiation, extensive disease or diffuse mammographic calcifications (range >30 mm), multifocal disease, unwillingness to undergo PM or clinically suspected inflammatory breast cancer. The exclusion criteria specified those PM patients without detailed information on surgical margin status.

We firstly reviewed the data with respect to the number of PMs and corresponding surgical procedures for the initial diagnosis of breast cancer from January, 2008 to June, 2015 at FUSCC. The latest data with percentage of PM patients from January, 2015 to June, 2015 in FUSCC remained the highest and was selected to analyse risk factors of positive margins after PM. Our study was approved by the independent ethical committee/ institutional review board of FUSCC. All patients were gave written informed consent before inclusion in this study.

### Surgical technique

PMs for breast cancer patients were performed by one experienced and qualified surgeon. PM consisted of excision of the tumour and the surrounding tissue at least 1 cm away from the tumour, extending from the subcutis to the pectoral fascia. We did not perform cavity shaving in PM patients in our centre. The decision regarding whether to pursue further surgical intervention for positive margins was left solely to the initial surgeon's discretion.

### Pathology examination for margins

The perpendicular inked margin technique, first proposed in 1986 [20] and accepted as a standard method for assessing margin status worldwide afterwards, has been used for margin status assessment at our centre since 2008. The breast specimen containing the tumour is oriented and inked with six different colours indicating each individual margin including superior, inferior, medial, lateral and anterior. The entire specimen is then sectioned perpendicular to the inked surface and submitted for microscopic examination. The exact distance between the tumour and each individually coloured inked margin is measured under a microscope and reported by the pathologist [21].

Margins were considered positive when the tumour involved the inked margin of the specimen in cases of invasive cancer or was within 1 mm of the inked margin for ductal carcinoma *in situ*.

**Figure 1:**
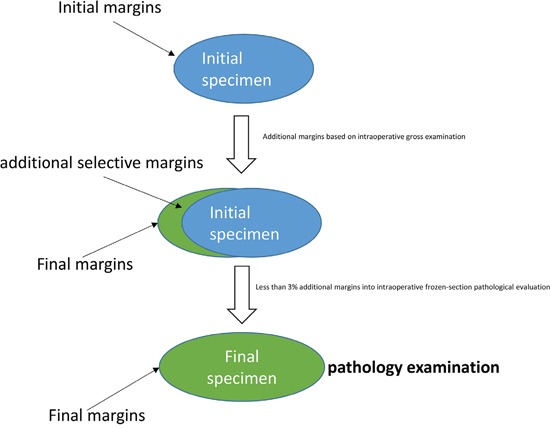
Surgical and pathological management of specimen and margins during and after PM

Additional margins were also obtained for pathological evaluation due to intraoperative gross examination by the surgeon or by a stand-by pathologist. Intraoperative frozen section evaluation for selective PM margins was not routinely applied in our centre except in a very small number of cases (less than 3%) when required by the surgeon (Figure 1).

### Data collection

The following demographic, clinical and pathological data for all the eligible patients was entirely extracted from the electronic medical records: age, preoperative mammographic calcifications, histological tumour size, histological grade, oestrogen receptor (ER), progesterone receptor (PR), human epidermal growth factor receptor-2 (HER-2), lymph nodal status, lymphovascular invasion status, and neo-adjuvant therapy. In our study, most patients with “mammographic calcifications” presented with the indicated malignant calcifications graded at least BI-RADS 4A on mammography, which were also pathologically validated before or after the PM. The detailed features of the calcifications were retrospectively analysed by a radiologist and a surgeon.

### Statistical analysis

All of the included variables were deemed categorical variables and analysed using Pearson's or Fisher's exact test in univariate analysis.

In multivariate analysis, logistic regression analysis was used to evaluate the association between margin positivity and possible factors including age, tumour size, mammographic calcifications, lymph nodal status, ER, HER-2, Ki-67, and lymphovascular invasion status.

All statistical analyses were performed using SPSS statistical software version 18.0, with p values reported as two-sided with an alpha of 0.05.

## SUPPLEMENTARY MATERIALS FIGURES AND TABLES


