# Supplementary material for: Low rate of positive margins and re-excision after partial mastectomy in highly selected breast cancer patients: A Chinese single-institution experience
Source: Oncotarget. 2017 Jan 17;8(7):12225–33. doi: 10.18632/oncotarget.14686 (PMC5355339; doi:10.18632/oncotarget.14686)
Supplement: Supplementary file 1 [file oncotarget-08-12225-s001.pdf]

Low rate of positive margins and re-excision after partial mastectomy in highly selected breast cancer patients: A Chinese single-institution experience

SUPPLEMENTARY TABLE

Supplementary Table 1: Number of patients undergoing PM and surgical treatment including PM and mastectomy from January 2010 to June 2015

Period	N (PMs)	N (total)	Rate
**Jan. 2010 to Dec. 2010**	332	1833	18.1%
**Jan. 2011 to Dec. 2011**	397	2240	17.7%
**Jan. 2012 to Dec. 2012**	399	2500	16.0%
**Jan. 2013 to Dec. 2013**	527	2969	17.8%
**Jan. 2014 to Dec. 2014**	708	3678	19.2%
**Jan. 2015 to June 2015**	408	1796	22.7%
